# The Use of a Liposomal Formulation Incorporating an Antimicrobial Peptide from Tilapia as a New Adjuvant to Epirubicin in Human Squamous Cell Carcinoma and Pluripotent Testicular Embryonic Carcinoma Cells

**DOI:** 10.3390/ijms160922711

**Published:** 2015-09-18

**Authors:** Yu-Li Lo, Hsin-Pin Lee, Wei-Chen Tu

**Affiliations:** 1 Department and Institute of Pharmacology, National Yang-Ming University, No. 155, Sec. 2, Linong Street, Taipei 112, Taiwan; 2 Department of Biological Sciences and Technology, National University of Tainan, Tainan 700, Taiwan; E-Mails: roselee6688@yahoo.com.tw (H.-P.L.); luyu2013@hotmail.com.tw (W.-C.T.)

**Keywords:** multidrug resistance, liposomes, reactive oxygen species, antimicrobial peptide, epirubicin, apoptosis

## Abstract

This study aims to explore the effects and mechanisms of hepcidin, a potential antimicrobial peptide from Tilapia, and epirubicin (Epi), an antineoplastic agent, on the generation of reactive oxygen species (ROS) and link the ROS levels to the reversal mechanisms of multidrug resistance (MDR) by epirubicin and hepcidin in human squamous cell carcinoma SCC15 and human embryonal carcinoma NT2D1 cells. The cells, pretreated with hepcidin, epirubicin, or a combination of these compounds in PEGylated liposomes, were used to validate the molecular mechanisms involved in inhibiting efflux transporters and inducing apoptosis as evaluated by cytotoxicity, intracellular accumulation, mRNA levels, cell cycle distribution, and caspase activity of this combination. We found that hepcidin significantly enhanced the cytotoxicity of epirubicin in liposomes. The co-incubation of epirubicin with hepcidin in liposomes intensified the ROS production, including hydrogen peroxide and superoxide free radicals. Hepcidin significantly increased epirubicin intracellular uptake into NT2D1 and SCC15 cells, as supported by the diminished mRNA expressions of MDR1, MDR-associated protein (MRP) 1, and MRP2. Hepcidin and/or epirubicin in liposomes triggered apoptosis, as verified by the reduced mitochondrial membrane potential, increased sub-G1 phase of cell cycle, incremental populations of apoptosis using annexin V/PI assay, and chromatin condensation. As far as we know, this is the first example showing that PEGylated liposomal TH1-5 and epirubicin gives rise to cell death in human squamous carcinoma and testicular embryonic carcinoma cells through the reduced epirubicin efflux via ROS-mediated suppression of P-gp and MRPs and concomitant initiation of mitochondrial apoptosis pathway. Hence, hepcidin in PEGylated liposomes may function as an adjuvant to anticancer drugs, thus demonstrating a novel strategy for reversing MDR.

## 1. Introduction

Cationic antimicrobial peptides (AMPs) can be found from prokaryotes to humans mainly in the innate immune system [1]. They demonstrate activities against diverse pathogens, including bacteria, viruses, fungi, mycoplasma, and parasites [2,3]. The positively-charged characteristics of AMPs allow them to penetrate and interact with anionic molecules on the surface of cancer cell membrane, such as phosphatidylserine and terminal sialic acids, including N-linked and O-linked glycans, thus causing cytotoxic membrane disruption to certain cancer types, but not to normal cells [4,5]. However, the fundamental mechanisms for AMP-mediated membrane collapse and tumor-specific cytotoxicity are poorly understood at present. Moreover, because of their size and cationic properties, AMPs are less vulnerable to proteolysis in serum and urine, thus making them prospective candidates for the treatment of intravesical tumor [6]. Accordingly, cecropins A and B have exhibited good therapeutic potential for the management of invasive bladder cancer with the benefit of restricted cytotoxicity to normal cells [7].

Hepcidin (TH) is isolated from tilapia and possesses a potent antimicrobial activity, particularly against *Escherichia coli* [8,9]. This AMP plays a critical role in regulating systemic iron balance [10]. Three hepcidin isoforms were found, namely TH1-5, TH2-2, and TH2-3 [8]. TH1-5, composed of 22 amino acids, shows anti-inflammatory, neuroprotective, antiviral, immunomodulatory, and anticancer activities [11]. TH1-5 was verified to function as an antiviral agent against Japanese encephalitis virus infection [11]. TH1-5 also augmented the inhibitory effect in transgenic TH1-5 zebrafish against bacterial infections and exhibited a good potential to treat infectious diseases [12]. Moreover, striking evidences have indicated that the outer membrane lipoprotein of Enterobacteriaceae was recognized by several cationic α-helical AMPs, thus enhancing the transmembrane permeability and the bactericidal activities of these AMPs [13]. Interestingly, TH1-5 decreased the proliferation of cervical cancer cells through inducing apoptosis at low concentrations and provoking necrosis at high concentrations in HeLa cells [14].

Many mechanisms have been found to be associated with multidrug resistance (MDR). Two commonly found MDR-related mechanisms are the upregulation of drug efflux transporters such as P-glycoprotein (P-gp, encoded by *MDR1*) and multidrug-resistance associated proteins (MRPs) and the simultaneous activation of many prosurvival pathways such as anti-apoptosis pathway [15,16]. The efflux of anticancer drugs by P-gp and MRPs may diminish the intracellular drug concentration. Inhibition of transporter proteins by MDR reversing agents does not always elevate potency of chemotherapy. The simultaneous amplification of apoptosis in cancer cells seems to be critical for successful MDR overturn. Therefore, suppressing anti-apoptosis factors such as Bcl-2 and inducing apoptosis factors such as Bax may allow programmed cell death and promote the therapeutic efficacy of anticancer drugs [17,18].

**Figure 1:**
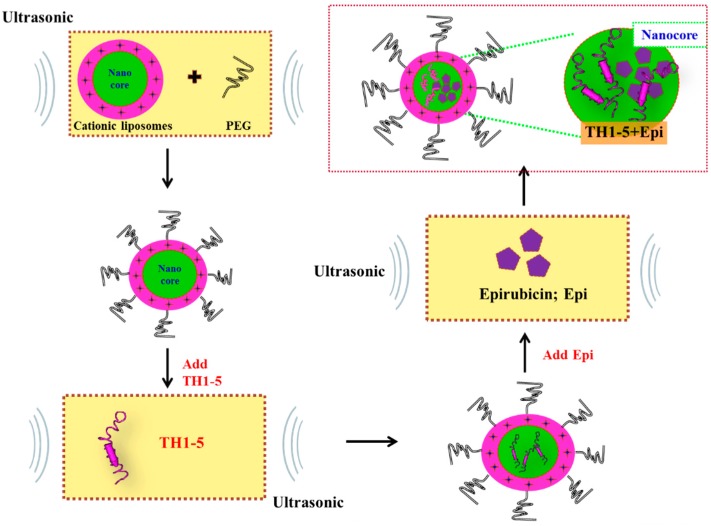
A schematic diagram of the formation of PEGylated liposomes containing epirubicin (Epi) and/or hepcidin 1-5 (TH1-5).

The clinical application of AMPs as MDR modulators is hampered because of low intrinsic selectivity for tumor cells and the high cost of manufacturing [19]. Furthermore, only few *in vivo* studies demonstrated the use of AMPs in tumors [5,20]. The development of PEGylated liposomes incorporating epirubicin, an anthracycline, and TH1-5, an AMP, may hold promise for reducing epirubicin efflux and intensifying the apoptosis induction effect of epirubicin. Hopefully, this combined use of TH1-5 and epirubicin incorporated in the PEGylated liposomal formulation might overcome traditional MDR mechanism(s) and augment the efficiency of epirubicin in human squamous cell carcinoma SCC15 and human pluripotent testicular embryonic carcinoma NT2/D1 (NTERA-2 cl.D1) cells. A schematic representation of the generation of PEGylated liposomes comprising Epi and/or TH1-5 is exhibited in Figure 1.

## 2. Results and Discussion

### 2.1. Results

#### 2.1.1. Determination of Encapsulation Efficiency, Particle Size, and Zeta Potential of PEGylated Liposomal TH1-5 or Epi

The encapsulation efficiency (%) of TH1-5 and Epi in PEGylated liposomes changed from 87.28% ± 1.89% for Lip-Epi+CHY to 89.17% ± 2.33% for Lip-Epi, as displayed in Table 1. These PEGylated liposomal preparations with or without TH1-5 and/or Epi were well-dispersed nanoparticles with sizes ranging from 93.12 ± 5.31 nm for Lip to 108.1 ± 4.67 nm for Lip-Epi+TH1-5, with a homogeneous polydispersity index about 0.1 (Table 1). In these liposomes, the mean zeta potential of Lip was 25.26 ± 2.88 mV (*n* = 4), indicating highly cationic property of this liposomal formulation (Table 1). As Epi was enclosed into liposomes, the zeta potential of Lip-Epi was marginally increased due to the cationic characteristic of Epi. When TH1-5 was encapsulated into liposomes, the zeta potential of these formulations additionally increased, possibly caused by the positive charges of TH1-5 (Table 1). The net zeta potential of these PEGylated liposomal formulations has demonstrated cationic characteristics, which might increase electrostatic interactions between these nanoparticles and anionic surface of tumor cells.

**Table 1:** Characteristics of liposomal formulations of TH1-5 and/or Epi (*n* = 4).

Formulations	Particle Size (nm)	Zeta Potential (mV)	PDI ^a^	EE% ^b^
Lip	93.12 ± 5.31	25.26 ± 2.88	0.101 ± 0.023	-
Lip-Epi	99.34 ± 2.26	26.32 ± 2.56	0.124 ± 0.027	91.26 ± 3.09
Lip-TH1-5	100.89 ± 2.67	27.78 ± 2.35	0.152 ± 0.087	88.32 ± 2.15
Lip-Epi+TH1-5	101.1 ± 4.67	30.66 ± 3.16	0.168 ± 0.089	87.28 ± 2.53

^a^ PDI, polydispersity index; ^b^ EE% (encapsulation efficiency) was calculated as the percentage of the amount of Epi (or TH1-5) in liposomes divided by the total amount of added Epi (or TH1-5).

#### 2.1.2. Epi and TH1-5 in PEGylated Liposomes Considerably Increased Epi Cytotoxicity

**Figure 2:**
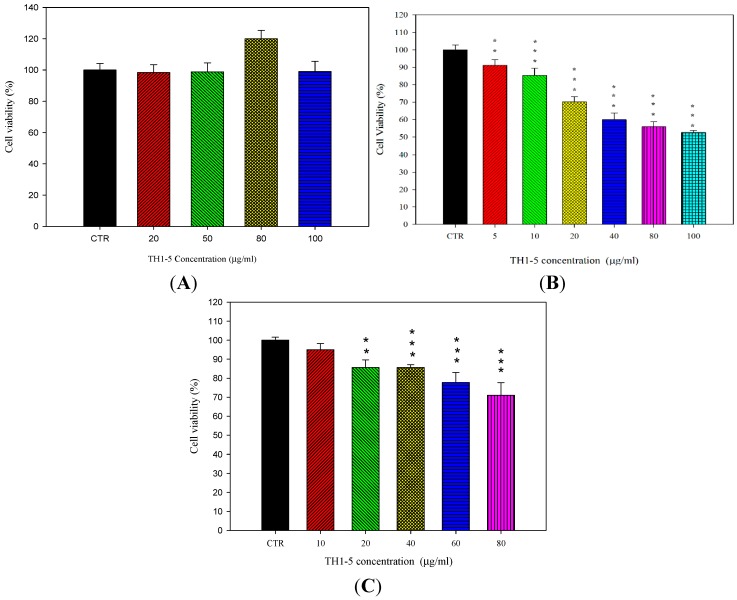
The effect of TH1-5 at different concentrations on the cell viability of (**A**) HeLa; (**B**) SCC-15; and (**C**) NT2D1 cells. Data are presented as means ± standard deviation (S.D.) from three independent experiments. Each experiment was conducted in triplicate. ** *p* < 0.01; *** *p* < 0.001 compared to the control (CTR).

**Figure 3:**
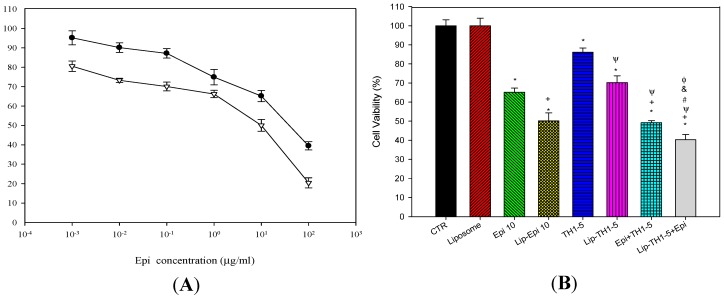

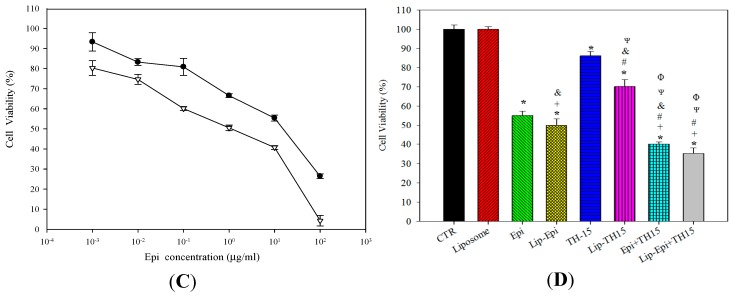
The effect of TH1-5 on the cytotoxicity of Epi in (**A**) SCC15 and (**C**) NT2D1 cells. ●: Epi alone; ▽: Epi plus TH1-5. In addition, the effect of different treatments on the cell viability of (**B**) SCC15 and (**D**) NT2D1 cells. Each experiment was conducted in triplicate. Data are presented as means ± S.D. from three independent experiments. * *p* < 0.05 compared to CTR; ^+^ *p* < 0.05 compared to Epi; ^Ψ^ *p* < 0.05 compared to TH1-5; ^#^ *p* < 0.05 compared to Epi+TH1-5; ^&^ *p* < 0.05 compared to Lip-Epi; ^Φ^ *p* < 0.05 compared with Lip-TH1-5.

The effect of TH1-5 at different concentrations on the cell viability of SCC15, NT2D1, and HeLa cells is shown in Figure 2. TH1-5 did not show significant cytotoxicity to HeLa cells (Figure 2A). However, after incubation with 10 μg/mL of TH1-5 for 24 h, the viability percentage of SCC15 cells was decreased to 85.32% ± 4.22% (Figure 2B); while after treatment with 20 μg/mL of TH1-5 for 24 h, the viability percentage of NT2D1 cells was reduced to 85.60% ± 4.05% (Figure 2C). Because our aim was to use TH1-5 as a MDR reversing agent to potentiate the cytotoxicity of Epi, we chose the concentration of TH1-5 with about 15% cytotoxicity as an adjuvant for the combined treatment with Epi. The combination of Epi and TH1-5 exhibited greater inhibition on the viability of SCC15 and NT2D1 cells than those of Epi alone (Figure 3A,C). Furthermore, as presented in Figure 3B,D, the combined treatment of PEGylated liposomal Epi and TH1-5 displayed more cytotoxicity to SCC15 and NT2D1 cells compared with those of free and liposomal Epi or TH1-5 (all *p* < 0.05). Lip-Epi+TH1-5 was verified to exhibit the superior potency to all the other formulations for inducing cytotoxicity on SCC15 and NT2D1 cells (all *p* < 0.05; Figure 3B,D). However, the viability percentages of HeLa cells did not decrease after addition of TH1-5 (Figure 2A) and Lip TH1-5 (data not shown) (*p* > 0.05). Consistently, the formulation of Lip-Epi+TH1-5 did not provide further improvement on the cytotoxicity of Lip-Epi to HeLa cells (*p* > 0.05; data not shown). We thus did not further investigate these formulations on HeLa cells. Instead, we verified the cytotoxic effect of TH1-5 on both SCC15 and NT2D1 cells and subsequently confirmed if TH1-5 and Epi in the PEGylated liposomal formulation might increase the efficacy of Epi on these two cell lines.

#### 2.1.3. PEGylated Liposomal Epi and TH1-5 Enhanced ROS Generation in SCC15 and NT2D1 Cells

**Figure 4:**
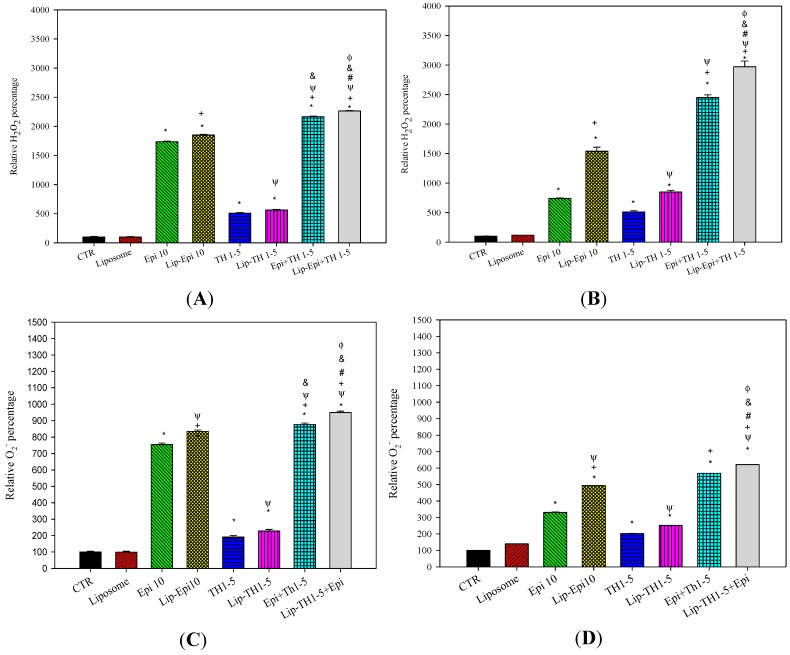
The effect of Epi and/or TH1-5 in free or liposomal formulations for 24 h on (**A**,**B**) hydrogen peroxide (H_2_O_2_) and (**C**,**D**) superoxide (O_2_^−^) production in (**A**,**C**) SCC15 and (**B**,**D**) NT2D1 cells. Means ± S.D. from three independent experiments are shown. In (**A**,**B**), mean DCF fluorescence intensity of cell control was normalized as 100%; In (**C**,**D**), mean EtBr fluorescence intensity of cell control was normalized as 100%. Data are presented as means ± S.D. from three independent experiments. * *p* < 0.05 compared to CTR; ^+^ *p* < 0.05 compared to Epi; ^Ψ^ *p* < 0.05 compared to TH1-5; ^#^ *p* < 0.05 compared to Epi+TH1-5; ^&^ *p* < 0.05 compared to Lip-Epi; ^Φ^ *p* < 0.05 compared with Lip-TH1-5.

Intracellular ROS levels, including H_2_O_2_ and O_2_^−^ levels, were measured by flow cytometry. Cell permeant probes 2′,7′-dichlorofluorescein diacetate (DCFH-DA) and dihydroethidium (DHE) were used to monitor the H_2_O_2_ and O_2_^−^ production, correspondingly. DCFH-DA and DHE are individually converted into fluorescent dichlorofluorescein (DCF) and ethidium bromide (EtBr) products inside the cells. The H_2_O_2_ levels showed that Epi or Lip-Epi produced noticeably higher amounts of H_2_O_2_ compared with TH1-5 or Lip-TH1-5, respectively, in both SCC15 and NT2D1 cells (Figure 4A,B). The relative intracellular H_2_O_2_ percentage was highest after the Lip-Epi+TH1-5 treatment among all the formulations used in this study (*p* < 0.05) (Figure 4A,B).

Moreover, the relative intracellular O_2_^−^ percentage also revealed the similar trend. SCC15 and NT2D1 cells incubated with free or liposomal Epi and/or TH1-5 have shown a remarkable change in O_2_^−^ production compared with the control (Figure 4C,D; all *p* < 0.05). The result also indicated that the Lip-Epi+TH1-5 formulation triggered the highest level of O_2_^−^ generation among all the treatments used in this study (*p* < 0.05; Figure 4C,D).

#### 2.1.4. PEGylated Liposomal Epi and TH1-5 Diminished the mRNA Levels of ABC Transporters

**Figure 5:**
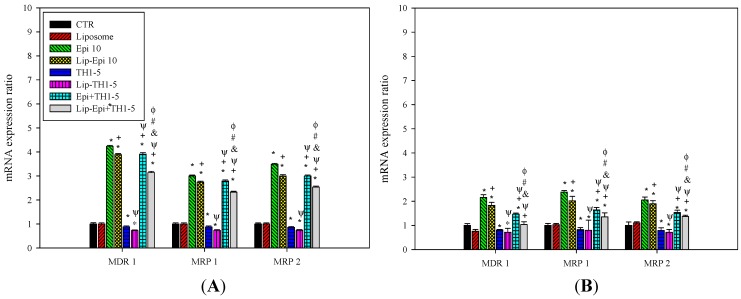
The effect of different treatments on the expression ratio of the MDR pump-related genes including MDR1, MRP1, and MRP2 in (**A**) SCC15 and (**B**) NT2D1 cells. Each experiment was conducted in triplicate. * *p* < 0.05 compared to CTR; ^+^ *p* < 0.05 compared to Epi; ^Ψ^ *p* < 0.05 compared to TH1-5; ^#^ *p* < 0.05 compared to Epi+TH1-5; ^&^ *p* < 0.05 compared to Lip-Epi; ^Φ^ *p* < 0.05 compared with Lip-TH1-5.

The mRNA expression levels of MDR1, MRP1, and MRP2 were measured using quantitative real-time PCR (qPCR). Epi and Lip-Epi both remarkably increased the mRNA levels of MDR1, MRP1, and MRP2 in SCC15 and NT2D1 cells (*p* < 0.05, Figure 5A,B), implying that Epi stimulated more ABC transporters to efflux Epi out and augmented acquired resistance. The mRNA expression ratios of MDR efflux proteins have been moderately decreased by TH1-5 and Lip-TH1-5 (*p* < 0.05, Figure 5A,B). The addition of Epi and TH1-5 in free or liposomal formulations all extensively diminished the respective mRNA levels of MDR1, MRP1, and MRP2 in comparison with the Epi or Lip-Epi treatment, separately (*p* < 0.05, Figure 5B). The mRNA expression level of MDR1 incubated with Lip-Epi+TH1-5 formulation was similar to the level of the control (*p* > 0.05) in NT2D1 cells, providing the significant induction of MDR1 by Epi or Lip-Epi in this cell line (Figure 5B). This finding implied that MDR1-mediated resistance is efficaciously circumvented by the addition of TH1-5 in the Lip-Epi formulation in NT2D1 cells (Figure 5B), whereas MRP1- or MRP2-associated Epi resistance was partially suppressed by Lip-Epi+TH1-5 treatment in other cases of SCC15 and NT2D1 cells (Figure 5A,B).

#### 2.1.5. PEGylated Liposomal TH1-5 Enhanced the Cellular Uptake of Epi into Cancer Cells

**Figure 6:**
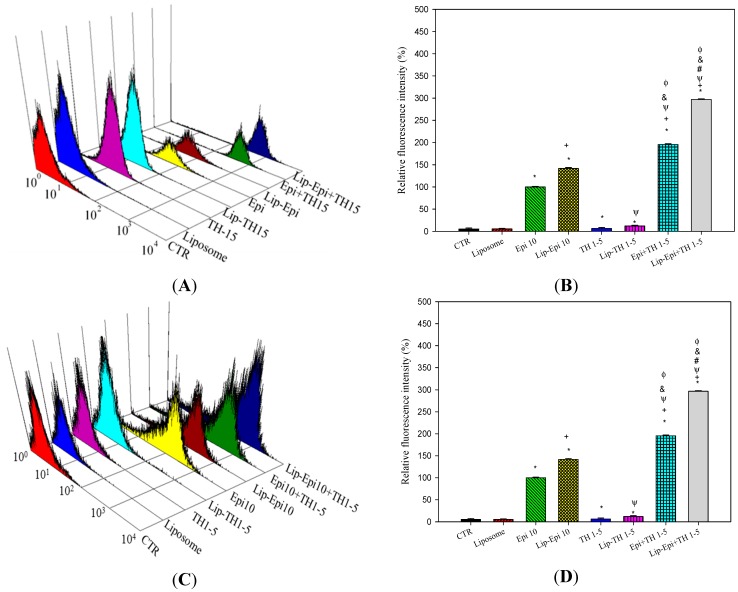
The effect of different treatments on the intracellular accumulation of fluorescent Epi in (**A**,**B**) SCC15 and (**C**,**D**) NT2D1 cells. The cells were pretreated with TH1-5 and/or Epi in free or liposomal formulations for 24 h. Three-dimensional view of cell number *versus* fluorescence intensity of epirubicin in (**A**) SCC15 or (**C**) NT2D1 cells is shown. The representative plots of flow cytometric analysis are displayed. In (**B**,**D**), the mean fluorescence intensity of epirubicin was normalized as 100%. Mean fluorescence intensity levels of other treatments were compared to the value of Epi. Data are presented as means ± S.D. from three independent experiments. * *p* < 0.05 compared to CTR; ^+^ *p* < 0.05 compared to Epi; ^Ψ^ *p* < 0.05 compared to TH1-5; ^#^ *p* < 0.05 compared to Epi+TH1-5; ^&^ *p* < 0.05 compared to Lip-Epi; ^Φ^ *p* < 0.05 compared with Lip-TH1-5.

The present result verified that TH1-5 in free and PEGylated liposomal formulations intensified the cellular uptake of Epi into SCC15 and NT2D1 cells after 24 h treatment (*p* < 0.05; Figure 6). This data further support the fact that reversing P-gp and MRPs using TH1-5 may explain the decrease in ABC transporter function and the corresponding improvement in Epi’s intracellular accumulation.

#### 2.1.6. Epi and TH1-5 Encapsulated in PEGylated Liposomes Decreased the Mitochondrial Membrane Potential of SCC15 and NT2D1 Cells

**Figure 7:**
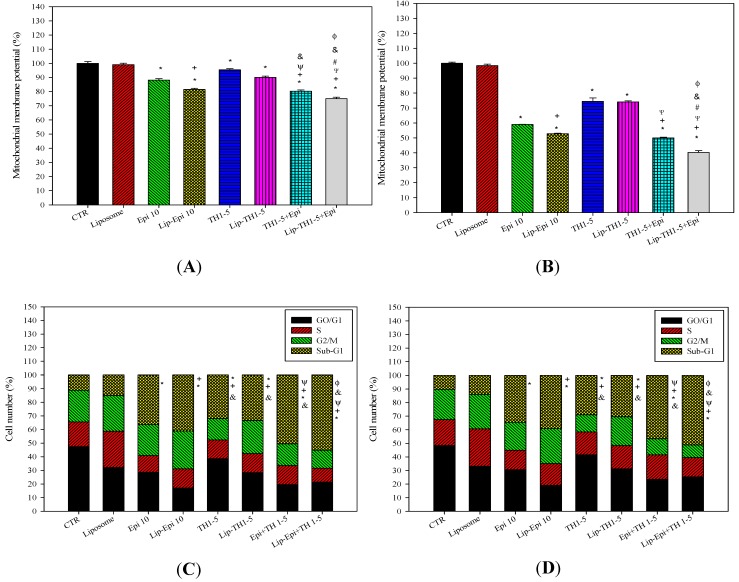
The effect of different treatments on the mitochondrial membrane potential of (**A**) SCC15 and (**B**) NT2D1 cells. Additionally, the effect of different treatments on the cell cycle distribution of (**C**) SCC15 and (**D**) NT2D1 cells. Data are presented as means ± S.D. from three independent experiments. * *p* < 0.05 compared to CTR; ^+^ *p* < 0.05 compared to Epi; ^Ψ^ *p* < 0.05 compared to TH1-5; ^#^ *p* < 0.05 compared to Epi+TH1-5; ^&^ *p* < 0.05 compared to Lip-Epi; ^Φ^ *p* < 0.05 compared with Lip-TH1-5.

A transmembrane fluorescent probe DiOC_6_ was used to detect the mitochondrial membrane potential difference (ΔΨ_m_) [21]. There was a significant decrease in ΔΨ_m_ after treatments of SCC15 and NT2D1 cells with Epi and/or TH1-5 for 24 h (Figure 7A,B). Epi and TH1-5 incorporated in PEGylated liposomes further diminished ΔΨ_m_. The formulation of PEGylated liposomal TH1-5 and Epi demonstrated a superior effect on ΔΨ_m_ reduction to all the other formulations used in this study.

#### 2.1.7. Liposomal or Free TH1-5 and/or Epi Treatment Remarkably Enhanced Apoptosis of SCC15 and NT2D1 Cells

When SCC15 and NT2D1 cells were incubated with Epi and/or TH1-5, they both displayed a traditional cell cycle phase distribution as measured by flow cytometry. The apoptotic cell sub-phase, as represented by the sub-G_1_ population, was significantly amplified after treatment with Epi, Lip-Epi, TH1-5, or Lip-TH1-5 for 24 h (Figure 7C,D). The percentage of the sub-G1 phase of cells after combined treatment of Epi and TH1-5 in free or liposomal formulation was significantly higher than those treatments with either TH1-5 or Epi alone with or without liposomes (all *p* < 0.05). Thus, TH1-5 was verified to function as an adjuvant to intensify the potency of Epi-provoked apoptosis in SCC15 and NT2D1 cells. 

**Figure 8:**
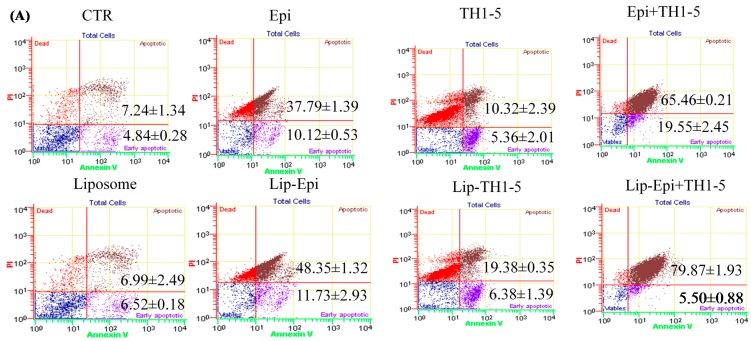

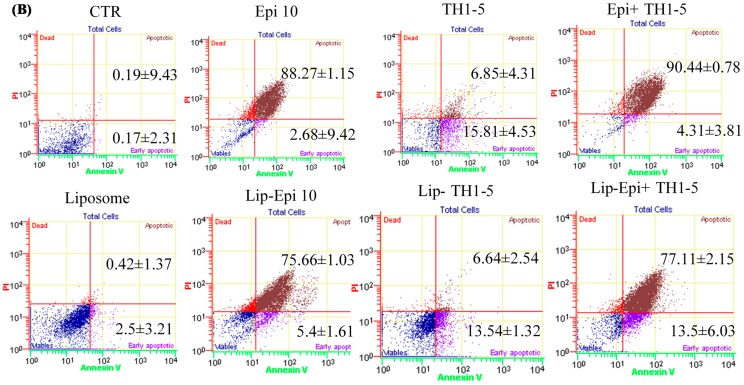
Quantitative analysis of cell apoptosis and necrosis induced by different treatments in (**A**) SCC15 and (**B**) NT2D1 cells. The cells were incubated with different treatments for 24h and stained with Annexin-V (AnnV) and propidium iodide (PI). Viable, apoptotic, and necrotic cells were then analyzed and quantified by a flow cytometer using an AnnV/PI staining kit. The percentage of early (AnnV^+^PI^−^) or late apoptotic (AnnV^+^PI^+^) are shown. Data are presented as means ± S.D. from three independent experiments.

During early stage of apoptosis, PS is exposed to the outer surface of the cell membrane, thus interacting with Annexin V, which can bind to fluorescent FITC. The early apoptotic cells expose PS and show Annexin V positive and PI negative. Nonetheless, the late apoptosis cells exhibit Annexin V and PI double positive, as displayed in Figure 8A,B. The results have demonstrated that when SCC15 and NT2D1 cells were incubated with Epi and/or TH1-5, they displayed a substantial increase in early and/or late apoptosis compared with the control. This was evident by the greater percentages of positive staining for Annexin V and/or PI (Figure 8A,B). The prominent rise in both early and late apoptosis percentages was most noticeable for the combined treatment of free Epi and TH1-5 in SCC15 cells (Figure 8A). Nevertheless, the PEGylated liposomal formulation of Epi and TH1-5 conspicuously moved the cell distribution to late apoptosis with a remarkable decline in the percentage of early apoptosis of SCC15 cells (Figure 8A). On the contrary, although Epi and TH1-5 also stimulated a pronounced increase in the percentage of late apoptosis, this value was reduced after the use of liposomal Epi and TH1-5 in NT2D1 cells. Accordingly, the population of early apoptosis was thus increased to a higher level after treatment with liposomal Epi and TH1-5 in NT2D1 cells (Figure 8B).

#### 2.1.8. PEGylated Liposomal Epi and TH1-5 Modified mRNA Expressions of p53, Bax, and Bcl-2

**Figure 9:**
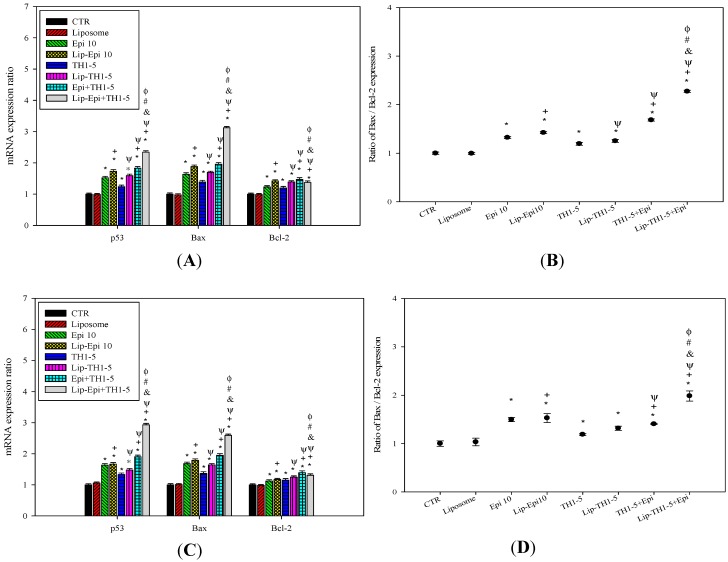
The effect of different treatments on the expression ratios of the apoptosis-related genes encoding Bax, Bcl-2, and p53 in (**A**) SCC15 and (**C**) NT2D1 cells, as measured by quantitative real-time PCR. Each experiment was conducted in triplicate. The effect of different treatments on the ratio of Bax: Bcl-2 mRNA expressions in (**B**) SCC15 and (**D**) NT2D1 cells. ●: ratio of Bax: Bcl-2 expression; * *p* < 0.05 compared to CTR; ^+^ *p* < 0.05 compared to Epi; ^Ψ^ *p* < 0.05 compared to TH1-5; ^#^ *p* < 0.05 compared to Epi+TH1-5; ^&^ *p* < 0.05 compared to Lip-Epi; ^Φ^ *p* < 0.05 compared with Lip-TH1-5.

The mRNA expression levels of p53, Bax, and Bcl-2 were assessed by quantitative PCR (Figure 9). Liposomal or free TH1-5 and/or Epi treatments all augmented Bax and p53 mRNA expressions, implying induction of apoptosis in both SCC15 and NT2D1 cells (Figure 9A,C; *p* < 0.05). Interestingly, all these treatments marginally increased the mRNA levels of Bcl-2 (Figure 9A,C; *p* < 0.05). However, Bax-to-Bcl-2 ratios of these treatments were moderately increased (Figure 9B,D; *p* < 0.05), indicating that Epi-mediated resistance has been partially reversed via the combined treatment with TH1-5, especially in the liposomal formulation. Lip-Epi+TH1-5 possessed the highest levels of mRNA expressions of Bax and p53, and Bax-to-Bcl-2 ratio among all the formulations used in this study.

#### 2.1.9. PEGylated Liposomal Epi and TH1-5 Changed the mRNA Expressions and Activity Levels of Caspases

**Figure 10:**
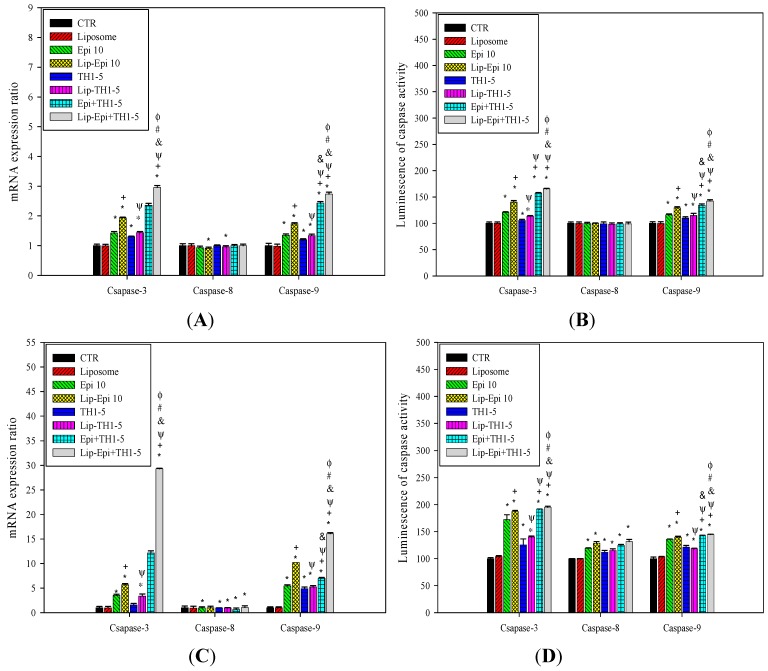
(**A**) The effect of different treatments on the expression ratio of the apoptosis-related genes encoding Caspase-3, Caspase-8, and Caspase-9 in (**A**) SCC15 and (**C**) NT2D1. The effect of different treatments on the activity levels of Caspase-3, Caspase-8, and Caspase-9 in (**B**) SCC15 and (**D**) NT2D1. The Caspase-Glo 3/7, Caspase-Glo 8, and Caspase-Glo 9 reagent was added directly to cells and incubated at room temperature before recording luminescence with a luminometer. Each experiment was conducted in triplicate. * *p* < 0.05 compared to CTR; ^+^ *p* < 0.05 compared to Epi; ^Ψ^ *p* < 0.05 compared to TH1-5; ^#^ *p* < 0.05 compared to Epi+TH1-5; ^&^ *p* < 0.05 compared to Lip-Epi; ^Φ^ *p* < 0.05 compared with Lip-TH1-5.

The Epi or TH1-5 treatments significantly increased the corresponding mRNA expressions and activity levels of caspases 3 and 9 in SCC15 and NT2D1 cells (Figure 10; *p* < 0.05). The combined Epi and TH1-5 treatment gave rise to more expressions and activity levels of caspases 3 and 9 (all with *p* < 0.05; Figure 10). Remarkably, the treatments of Lip-Epi+TH1-5 further amplified the mRNA levels and activity levels of caspases 3 and 9, respectively (all with *p* < 0.05). Our result thus verified that TH1-5 might boost the sensitivity of SCC15 and NT2D1 cells to apoptosis activated by Epi. All these treatments had negligible effects on the mRNA expression and activity level of caspase 8, indicting the marginal contribution of extrinsic apoptotic pathway. The noteworthy increases in the mRNA expression and activity levels of caspase 3 and 9 further established the connection of intrinsic mitochondrial pathway to the apoptosis aggravated by Epi and/or TH1-5.

#### 2.1.10. FREE or Liposomal TH1-5 and/or Epi Treatments Gave Rise to Morphological Changes Observed by Fluorescence Microscope

**Figure 11:**
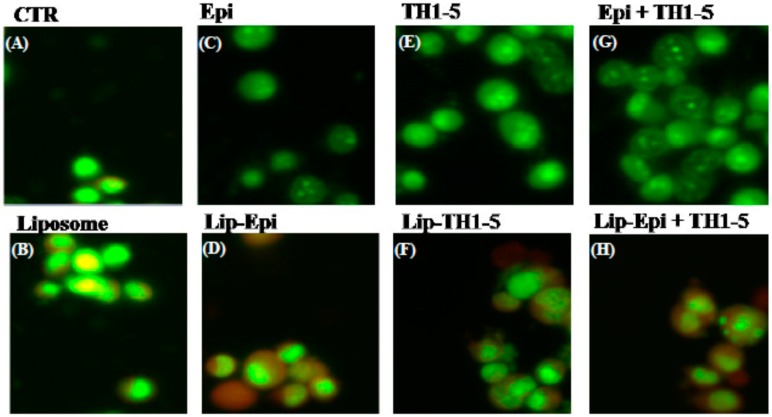
Nuclear chromatin condensation in NT2D1 cells by different treatments; after treatment with TH1-5 and/or Epi in free or liposomal formulations for 24 h, cells were mixed with acridine orange (AO). Apoptosis cells were distinguished through chromosomes using Nikon fluorescence microscopy. The images were visualized using an inverted microscope (Eclipse TS-100) equipped with a fluorescence image capture device (C-SHG; Nikon) controlled with an Image-Pro Plus software. (**A**) CTR; (**B**) Liposome; (**C**) Epi; (**D**) Lip-Epi; (**E**) TH1-5; (**F**) Lip-TH1-5; (**G**) Epi+TH1-5; (**H**) Lip-Epi+TH1-5.

**Figure 12:**
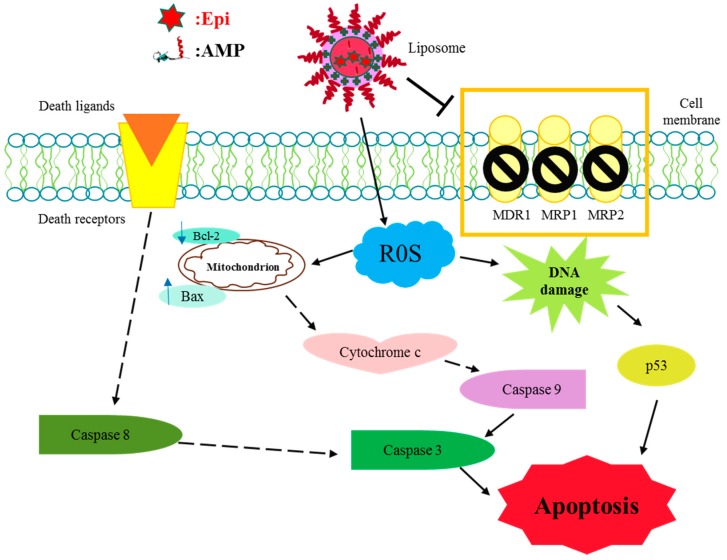
Proposed pathway for reversing pump and non-pump MDR in SCC15 and NT2D1 cells.

The viable NT2D1 control cells exhibited bright green fluorescence, as displayed using a fluorescence microscopy (Figure 11A). The cells exposed to empty liposomes (no Epi and Th1-5 incorporation) showed the same characteristics as the control (Figure 11B). When NT2D1 cells were treated with 20 μg/mL TH1-5 and/or 10 μg/mL Epi for 24 h, obvious chromatin condensation was found in the nucleus of these cells (Figure 11C–H). SCC15 cells also demonstrated the similar phenomena (Data not shown). The fluorescence microscopy images (Figure 11) further reinforced other apoptotic evidence in the present study (Figure 7, Figure 8, Figure 9 and Figure 10). Thus, we corroborated that TH1-5 and/or Epi in free or liposomal formulation caused death of SCC15 and NT2D1 cells mainly via apoptosis-triggering mechanism. The anticipated pathways for circumventing ABC efflux proteins and provoking apoptosis using PEGylated liposomal Epi and/or TH1-5 in SCC15 and NT2D1 cells are illustrated in Figure 12.

### 2.2. Discussion

TH1-5 is found most in the liver and kidney of *Oreochromis mossambicus* [8]. Evidence from *in vivo* and *in vitro* studies has supported that TH1-5 has antiviral, immunomodulatory, anti-inflammatory, and neuroprotective activities [11]. Synthesized TH1-5, including 22 amino acids, inhibited the proliferation of HeLa cells through inducing necrosis at high concentrations and triggering apoptosis at low concentrations [14]. Furthermore, TH1-5 has been shown to downregulate the expressions of caspase-7, Bcl-2, interleukin (IL)-6, -8, and interferon (IFN)-γ, and upregulate IL-2 and calpain 5 in HeLa cells [14]. Nevertheless, this AMP upregulated IL-8 and cathepsin G in human fibrosarcoma HT1080 cells. These findings indicate that TH1-5 initiates inflammatory pathways in HeLa cells, but not in HT1080 cells [14]. Moreover, hepcidin was downregulated in human hepatocellular carcinoma HepG2 cells. Treatment with trichostatin A increased the expression of hepcidin in HepG2 cells, suggesting that inhibition of histone deacetylation might upregulate hepcidin expression with a cancer intervention potential [22].

TH1-5 possesses α-helix in its structure and carries three positive charges and 59% hydrophobic residues, thus displaying amphiphilic properties [8]. Cationic AMPs such as TH1-5 may interact with anionic glycosaminoglycans on cancer cell surface and these interactions may cause cancer cell death due to membrane collapse [4]. AMPs are thus less attracted to normal cell membranes comprising neutral phospholipids and cholesterols [23]. The negatively charged molecules are at least partially responsible for the selective lytic effect of AMPs on cancer cell membranes that may extend to the disruption and swelling of mitochondria, causing cytochrome c release and apoptosis induction [24,25]. However, AMPs, e.g., TH1-5, have traditional drawbacks, including poor pharmacokinetic characteristics and possible toxicity to erythrocytes, which may restrain their clinical application.

A potent strategy to overwhelm these limits is to incorporate them into appropriate dosage forms, such as liposomes. In this study, the deliberate delivery system consisting of epirubicin and TH1-5 in a PEGylated liposomal formulation has been demonstrated to decrease the efflux of epirubicin and increase the sensitivity of SCC15 and NT2D1 cells to apoptosis induced by epirubicin. We found that TH1-5 significantly enhanced cytotoxicity of epirubicin in free or liposomal formulations in these two cancer cell lines (Figure 3). The co-incubation of epirubicin and/or TH1-5 in liposomes in free or liposomal formulations increased the ROS levels, including hydrogen peroxide and superoxide free radicals in both SCC15 and NT2D1 cells (Figure 4). The combined treatment of TH1-5 and epirubicin in free or liposomal formulations significantly decreased the mRNA levels of MDR1, MRP1, and MRP2, which are markedly increased by single epirubicin or liposomal epirubicin treatment (Figure 5). Accordingly, these combined treatments intensified the intracellular epirubicin accumulation in NT2D1 and SCC15 cells (Figure 6). The reversal of MDR by liposomal epirubicin and TH1-5 against pump resistance genes was thus verified and discussed as follows.

Our results showed that epirubicin in free or liposomal formulations improved the intracellular ROS levels and amplified the expressions of ABC transporters. Free epirubicin usually passively diffuses into cancer cells and liposomal epirubicin enters cells via endocytosis. Nevertheless, some of the influxed epirubicin is actively pumped out by MDR transporters, thus consuming the energy provided by ATP. The remuneration of ATP via mitochondrial oxidative phosphorylation generates ROS, leading to oxidative stress. Such ROS stress induced by epirubicin treatment further provoked the expressions of P-gp and MRPs to pump epirubicin out of cancer cells, thus augmenting the acquired MDR, which were consistent with the earlier studies [16,26,27].

However, the combined treatment of free or liposomal TH1-5 and epirubicin potentiated intracellular ROS generation, but decreased the expression of P-gp and MRPs, which were originally induced by epirubicin or liposomal epirubicin treatment alone. This phenomenon was in accordance with some other previous studies displaying a negative correlation between ROS levels and ABC transporter expressions [16,28,29]. Our findings indicate that Epi-triggered resistance is abrogated at various degrees by adding TH1-5 into the free and liposomal formulations via H_2_O_2_ and O_2_^−^ production to reduce the mRNA expressions of MDR1, MRP1, and MRP2.

Interestingly, it has been reported that AMPs, including psacotheasin and pleurocidin, initiate apoptosis via intracellular ROS generation, especially cytotoxic hydroxyl radicals, thus inducing oxidative stress [30,31,32]. Plasma membrane depolarization and externalization of phosphatidylserine on the outer surface confirm apoptotic progress at early stages [6]. The concurrent release of cytochrome c and mitochondrial dysfunction result in the production of pro-apoptotic factors and caspase activation [32], and thus further verify the occurrence of apoptosis-mediated cell death. In addition, quinacrine, a 9-aminoacridine derivative, is a therapeutic agent used against malaria. It has been found to trigger apoptosis via oxidative stress in human leukemia K562 cells [33]. Quinacrine induced ROS production, activated p38 MAPK, and inhibited ERK/c-Jun, leading to mitochondrial depolarization and suppression of Bcl-2 and Bcl2l1 expressions in K562 cells [33]. Moreover, plumbagin, a vitamin K3 analog and a pro-oxidant, increased ROS generation, enhanced caspase activity, caused mitochondrial dysfunction, and thus provoked apoptosis in T-cell lymphoma cell lines [34]. The accompanied cytochrome c release and higher FasL and Bax expressions as triggered by plumbagin treatment were mediated via JNK signaling activation [34].

During the process of aerobic respiration, mitochondria produce ATP, thus generating ROS as by-products of oxidative phosphorylation. The accumulated oxidative stress triggered by the co-treatment of TH1-5 and/or epirubicin in PEGylated liposomes then induced apoptosis, as indicated by mitochondrial membrane potential reduction via DiOC6 staining (Figure 7A,B), increased sub-G1 phase (Figure 7C,D) via PI staining, exposure of phosphatidylserine on the outer surface via Annexin V staining (Figure 8), and chromatin condensation via AO staining (Figure 11) in the current study. These treatments significantly raised the mRNA expressions of p53 and Bax, and the ratio of Bax/Bcl-2 (Figure 9). These formulations also substantially elevated the mRNA expression and activity levels of caspase 3 and caspase 9 (Figure 10). Consistently, evidence has suggested that low concentrations of TH1-5 effectively decreased the growth of cervical cancer cells through apoptosis induction in HeLa cells [14]. All our results have verified the apoptosis-provoking effect of epirubicin on both SCC15 and NT2D1 cells, which was intensified by the co-treatment of TH1-5, particularly in the PEGylated liposomal formulation. The inhibition of MDR by liposomal epirubicin and TH1-5 against non-pump resistance genes via apoptosis induction was thus confirmed.

## 3. Experimental Section

### 3.1. Materials

The amino acid sequence of TH1-5 is GIKCRFCCGCCTPGICGVCCRF-NH2. TH1-5 was modified into an amidated C-terminus and synthesized by Genesis Biotech (Taipei, Taiwan) at >98% purity. This AMP was provided by Dr. J.Y. Chen, Marine Research Station, Institute of Cellular and Organismic Biology, Academia Sinica. Epirubicin (Pharmorubicin) was bought from Pfizer Inc. (New York, NY, USA). Polyethylene glycol 6000 (PEG6000) was obtained from Sigma-Aldrich (St. Louis, MO, USA). DOTMA and DOPE were purchased from Avanti Polar Lipids, Inc. (Alabaster, AL, USA). All cell culture medium and reagents were obtained from Promega (Madison, WI, USA), Invitrogen (Carlsbad, CA, USA), Gibco BRL (Grand Island, NY, USA), or Hyclone (Logan, UT, USA). All other chemical reagents were bought from either Merck (Darmstadt, Germany) or Sigma-Aldrich (St. Louis, MO, USA).

### 3.2. Cell Culture

Human cervical cancer HeLa, SCC15, and NT2D1 cell lines were obtained from the Food Industry Research and Development Institute (Hsinchu, Taiwan). These cells were maintained in Dulbecco’s modified Eagle’s medium (DMEM; Gibco) supplemented with 10% fetal bovine serum (Hyclone, Logan, UT, USA) and 1% streptomycin/penicillin (Hyclone). The culture was incubated in a humidified atmosphere of 5% CO_2_ at 37 °C.

### 3.3. Preparation of PEGylated Cationic Liposomal Formulations

Eight groups of treatments were prepared: control (CTR), empty liposome (Lip), Epi, liposomal Epi (Lip-Epi), TH1-5, liposomal TH1-5, Epi plus TH1-5, and liposomal Epi plus TH1-5. We performed these preparations as adjusted from Li and Huang’s report [35] and our previous study [36]. The thin film hydration method was used to prepare small PEGylated cationic unilamellar liposomes comprising DOTMA and DOPE. In short, 1 mg/mL each of DOTMA and DOPE (1:1 *w*/*w*) were heated to above lipid phase transition temperature. These lipids were then coated with PEG6000 (at a 1:1 molar ratio of PEG and DOPE) and maintained at 50 °C water bath for 2 h. The coating with PEG chains was ensured by further incubating the mixture under 25 °C ultrasonication for 10 min. Epi and TH1-5 were then incorporated into the PEGylated liposomes by ultrasonication at 25 °C for 30 min.

### 3.4. Determination of Size Distribution, Zeta Potential, and Encapsulation Efficiency (EE%)

The size distribution and zeta potential of liposomes were measured using an ELSZ-2000 dynamic light scattering system (Otsuka Electronics Co., Ltd., Osaka, Japan) at 25 °C. Data were analyzed from four individual measurements.

Additionally, free Epi and TH1-5 were separated from the encapsulated PEGylated liposomes by filtration and centrifugation at 15,000 rpm for 20 min (4 °C) using an Amicron Ultra-4 Centrifuge Filter (10,000 WCO, Millipore Corp., Billerica, MA, USA). TH1-5 in the filtrate was monitored by a Nanodrop spectrophotometer (Labtech, Ringmer, UK). Epi in the filtrate was analyzed by HPLC [37]. The HPLC system is composed of a L7100 pump (Hitachi, Tokyo, Japan), an autosampler (Primaide 1210), a LiChrospher column (25 cm long, 4 mm inside diameter; Merck), and a L2400 UV detector (Hitachi). The mobile phase was made of methanol and water (75:25, *v*/*v*). The flow rate was 1.2 mL/min and the detection wavelength was fixed at 254 nm. Each experiment was conducted in quadruplicate. EE% was computed by Equation (1):

*EE%* = [(*W*_e_ − *W*_f_)/*W*_e_] × 100% (1)

where *W*_e_ is the weight of added Epi (or TH1-5) and *W*_f_ is the weight of Epi (or TH1-5) in the filtrate.

### 3.5. Cell Viability Assay 

Six thousand each of HeLa, CT-26, SCC15, and NT2D1 cells were cultured in 96-well plates and incubated with the respective eight groups of treatments as mentioned above for 24 h. The cells were added with 0.2 mg/mL MTT (3-(4,5-dimethylthiazol-2-yl)-2,5-diphenyltetrazolium bromide; Sigma, St. Louis, MO, USA) and maintained for another 4 h. Dimethyl sulfoxide (DMSO, 100 μL) was added into each well to dissolve the formazan. The absorbance value was measured using an MRX microplate reader (Dynatech Laboratories Inc., Chantilly, VA, USA) at the wavelength of 540 nm. The detected OD_540_ for different treatments was changed into the cell number based on the standard curve. Relative cell viability (%) was calculated by dividing the number of cells treated with each group by the number of cell control (medium alone). Data were analyzed from three individual measurements.

### 3.6. Measurement of Intracellular Hydrogen Peroxide and Superoxide Levels

Two hundred thousand cells/well of individual cell lines were incubated in six-well plates and kept an overnight seeding. The cells were then exposed to Epi (10 μg/mL) and/or TH1-5 (20 μg/mL) with or without PEGylated liposomes for 24 h. The mixture was then incubated in the dark with 2′,7′-dichlorofluorescein diacetate (DCFH-DA) (20 μM) or dihydroethidium (DHE) (5 μM) at 37 °C for 30 min. The cells were then harvested and directly analyzed using a flow cytometer (Cell Lab Quanta SC MPL (abbreviated as Quanta SC); Beckman Coulter, Fullerton, CA, USA). The generated fluorescent dichlorofluorescein (DCF) and ethidium bromide (EtBr) were detected using an excitation at wavelength of 488 nm and the emission of fluorescence was monitored at 525 and 575 nm, separately. Data collection and computation were achieved using commercial software (Quanta SC) [29].

### 3.7. Real-Time Quantitative PCR of MDR1, MRP1, MRP2, Bax, Bcl-2, and Caspases

Various cell lines were cultured with different treatments of eight groups for 24 h. RNA was extracted using the Total RNA Extraction Miniprep System (Viogene, Taipei, Taiwan). The RNA amounts were measured using a Nanodrop spectrophotometer (Labtech, Ringmer, UK). RNA was reversely transcribed into cDNA using a high-capacity RNA-to-cDNA kit (Applied Biosystems, Foster City, CA, USA). The gene-specific primer sequences of MDR1, MRP1, MRP2, Bcl-2, and Bax, as well as caspases 3, 8 and 9 were demonstrated in our previous paper [36]. Glyceraldehyde-3-phosphate dehydrogenase (GAPDH) was set as an internal control. Quantitative PCR was run using the StepOne Real-Time PCR system (Applied Biosystems) and SYBR Green PCR Master Mix (Applied Biosystems). The gradient program was performed as follows: denaturation at 95 °C for 10 min, 40 cycles of 95 °C for 15 s, and 60 °C for 1 min. Each experiment was done in triplicate and normalized to the GAPDH level. The gene expression ratio was computed as the mRNA expression levels of a specific gene divided by the levels of cell control.

### 3.8. Functional Assay of ABC Transporters

After pretreatment with eight different formulations at 37 °C for 24 h, the cells at a density of 2 × 10^5^ cells/well were collected. The cellular uptake of Epi in SCC15 and NT2/D1 cells was then measured by fluorescence intensity analysis using a flow cytometer [16]. Data acquisition and assessment were carried out using commercial software (Quanta SC). All measurements were performed in triplicate.

### 3.9. Determination of Mitochondrial Membrane Potential with 3,3′-Dihexyloxacarbocyanine Iodide (DiOC_6_)

After overnight seeding of 2 × 10^5^ cells/well, the cells were exposed to Epi (5 μg/mL) and/or TH1-5 (5 μg/mL) with or without PEGylated liposomes for 24 h. The cells were incubated with 10 μM DiOC_6_ at 37 °C for 30 min and harvested. DiOC_6_ was excited at 488 nm and the fluorescence was instantaneously analyzed with a 525 nm (FL-1) band pass filter using a Quanta SC flow cytometer.

### 3.10. Cell Cycle Analysis

After incubation of 2 × 10^5^ cells/well with eight different formulations for 24 h, the cells were collected after centrifugation and mildly fixed with 70% ice-cold ethanol overnight at −20 °C. The fixed cells were stained with 1 mg/mL propidium iodide (PI) for 30 min in the dark and then immediately analyzed by flow cytometry. The following cell cycle distribution was observed: the sub-G1 phase for apoptotic cells with hypodiploid DNA content (<2 n), G0/G1 phase for growing diploid cells (DNA content: 2 n), S phase for replicating cells, and G2/M phase for diploid cells with replicated DNA (DNA content: 4 n).

### 3.11. Annexin V FITC Apoptosis Detection Assay

The Annexin V FITC Apoptosis Detection Kit was obtained from Roche (Cambridge, MA, USA). After overnight seeding of cells (2 × 10^5^ cells/well), the cells are exposed to Epi (5 μg/mL) and/or TH1-5 (20 μg/mL) in the presence and absence of PEGylated liposomes for 24 h. Staining was accomplished using Annexin V-propidium iodide (PI) labeling solution for 15 min at room temperature in the dark. The harvested cells were measured using a flow cytometer (Quanta SC). Data attainment and calculation were carried out using commercial software (Quanta SC). Early apoptotic cells with intact cell membranes expose phosphatidylserine and are bound to Annexin V-FITC (Annexin V positive, PI negative). When cells perform late apoptosis, they are Annexin V-FITC and PI positive. However, living cells are found in FITC^−^/PI^−^ quadrant; while necrotic cells in FITC^−^/PI^+^ quadrant.

### 3.12. Caspases 3, 8, and 9 Activity Assay

After treatment of 2 × 10^5^ cells/well with eight different formulations for 24 h, the cell pellets were collected and redispersed in medium. The cell suspension was disintegrated with lysis buffer, composed of 50 mM Tris–HCl, 10 mM EDTA, 0.5% Triton X-100, 0.5 mg/mL Proteinase K. Caspase 3, 8 and 9 activities were monitored using luminescence-based Caspase-Glo 3/7, 8 and 9 Assay Kits (Promega), separately. Fifty microliters of the cell lysate was mixed with an equal volume of caspase 3, 8 and 9 reagents at 25 °C for 30 min, respectively. These reagents include the respective luminogenic caspase 3, 8 and 9 substrates. Formed luminescence levels of aminoluciferin were detected using a luminometer (MiniLumat LB9506, Berthold Technologies, Bad Wildbad, Germany).

### 3.13. Chromatin Condensation Imaged by Fluorescence Microscopy

After overnight seeding of 2 × 10^5^ cells/well, the cells were harvested, stained with acridine orange (AO; 10 mg/mL; Sigma), and observed under an inverted microscope (Eclipse TS-100, Nikon Co., Tokyo, Japan). This microscope was supplied with a fluorescence image capture device (C-SHG, Nikon) and imaged using an Image-Pro Plus software (Media Cybernetics, Inc., Bethesda, MD, USA).

### 3.14. Statistical Analyses

All data are expressed as means ± SD for the indicated number of separate experiments. Student’s *t*-test was used to analyze differences between two treatment groups. Differences were considered to be significant at *p* < 0.05.

## 4. Conclusions

Collectively, this is the first study to display that PEGylated liposomal epirubicin and TH1-5 lead to cell death in human squamous carcinoma and pluripotent testicular embryonic carcinoma cells through the reduced epirubicin efflux via the TH1-5-mediated inhibition of ABC transporters and the caspase-dependent activation of the intrinsic mitochondrial pathway of apoptosis triggered by epirubicin in the liposomal formulation. As a novel adjuvant to diminish chemotherapy dosage and the corresponding side effects and to improve the therapeutic efficacy of clinically available anticancer drugs, TH1-5 may bypass the existing resistance mechanisms to the current antineoplastic agents and exhibit multifunctional potency in MDR-related pathways.
